# Soluble usokinase plasminogen activator receptor as a useful biomarker to define advent of sepsis in patients with multiple injuries

**DOI:** 10.1186/cc10631

**Published:** 2012-03-20

**Authors:** M Patrani, M Paraschos, M Georgitsi, E Giamarellos-Bourboulis, K Mandragos

**Affiliations:** 1 Korgialeneion Benakeion Hospital, Athens, Greece; 2 University of Athens, Medical School, Athens, Greece

## Introduction

Soluble usokinase plasminogen activator receptor (suPAR) has been considered a useful biomarker to define prognosis in patients with sepsis [1]. The present study aimed to define the kinetics of suPAR during the physical course of patients with multiple injuries.

## Methods

A total of 62 patients were enrolled. All patients were bearing: multiple injuries necessitating ICU admission with an injury severity score (ISS) more than 8; and systemic inflammatory response syndrome. Patients with infections upon ICU admission were excluded from the study. Peripheral venous blood was sampled within the first 24 hours after ICU admission. Blood sampling was repeated within the first 24 hours upon advent of sepsis. suPAR was measured in serum by an enzyme immnunoassay.

## Results

Mean ISS of patients was 14.6. Median suPAR upon ICU admission was 3.74 ng/ml (range: 1.57 to 16.77 ng/ml). No correlation was found between ISS and suPAR. Sepsis was presented in 27 patients. Median suPAR upon sepsis diagnosis was 7.05 ng/ml (range: 2.18 to 32.51 ng/ml) (*P *< 0.0001 compared with ICU admission). This change corresponded to median increase of 57.81%.

## Conclusion

The presented findings show that measurement of serum suPAR may help diagnosis of sepsis presenting in patients with multiple injuries.
